# A Generative Adversarial Network to Synthesize 3D Magnetohydrodynamic Distortions for Electrocardiogram Analyses Applied to Cardiac Magnetic Resonance Imaging

**DOI:** 10.3390/s23218691

**Published:** 2023-10-24

**Authors:** Maroua Mehri, Guillaume Calmon, Freddy Odille, Julien Oster, Alain Lalande

**Affiliations:** 1 Epsidy, 54000 Nancy, France; maroua.mehri@epsidy.com (M.M.); guillaume.calmon@epsidy.com (G.C.); 2 Ecole Nationale d’Ingénieurs de Sousse, LATIS-Laboratory of Advanced Technology and Intelligent Systems, Université de Sousse, 4023 Sousse, Tunisia; 3 IADI-Imagerie Adaptative Diagnostique et Interventionnelle, Inserm U1254, Université de Lorraine, 54000 Nancy, France; julien.oster@inserm.fr; 4 CIC-IT 1433, Inserm, CHRU de Nancy, Université de Lorraine, 54000 Nancy, France; 5 ICMUB Laboratory, CNRS 6302, University of Burgundy, 21000 Dijon, France; alain.lalande@u-bourgogne.fr; 6 Department of Medical Imaging, University Hospital of Dijon, 21079 Dijon, France

**Keywords:** data synthesis, electrocardiogram, generative adversarial network, cardiac magnetic resonance imaging, magnetohydrodynamic effect, R-peak detection

## Abstract

Recently, deep learning (DL) models have been increasingly adopted for automatic analyses of medical data, including electrocardiograms (ECGs). Large, available ECG datasets, generally of high quality, often lack specific distortions, which could be helpful for enhancing DL-based algorithms. Synthetic ECG datasets could overcome this limitation. A generative adversarial network (GAN) was used to synthesize realistic 3D magnetohydrodynamic (MHD) distortion templates, as observed during magnetic resonance imaging (MRI), and then added to available ECG recordings to produce an augmented dataset. Similarity metrics, as well as the accuracy of a DL-based R-peak detector trained with and without data augmentation, were used to evaluate the effectiveness of the synthesized data. Three-dimensional MHD distortions produced by the proposed GAN were similar to the measured ones used as input. The precision of a DL-based R-peak detector, tested on actual unseen data, was significantly enhanced by data augmentation; its recall was higher when trained with augmented data. Using synthesized MHD-distorted ECGs significantly improves the accuracy of a DL-based R-peak detector, with a good generalization capacity. This provides a simple and effective alternative to collecting new patient data. DL-based algorithms for ECG analyses can suffer from bias or gaps in training datasets. Using a GAN to synthesize new data, as well as metrics to evaluate its performance, can overcome the scarcity issue of data availability.

## 1. Introduction

**Figure 1:**
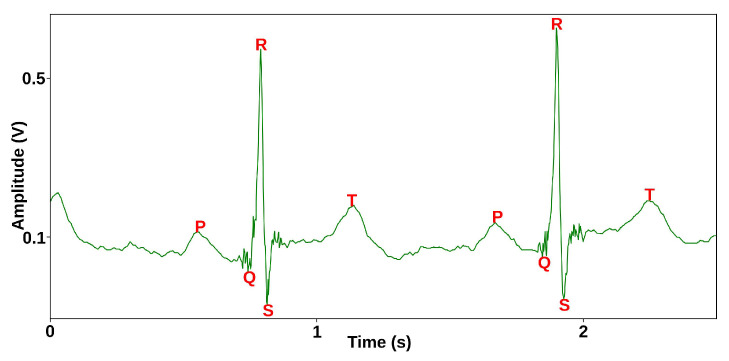
Two cardiac cycles showing key ECG features in lead II. P: atrial depolarization; Q: anteroseptal activation; R: ventricular depolarization; S: posterobasal activation; T: ventricular repolarization.

An electrocardiogram (ECG) is a recording of the electrical activity of the heart that is used by clinicians when a cardiovascular disease is suspected. It comprises a number of temporal signals, called leads. The 12-lead ECG is a widely used and studied cardiology standard. Features of interest include P waves, QRS complexes, T waves, and the onset or offset times of each feature (see Figure 1) [1]. A QRS complex happens about 100 msec before cardiac systole. In cardiac magnetic resonance imaging (MRI), R-peaks are used to trigger the acquisition of k-space data. The timely and accurate detection of R-peaks directly impacts image quality [2].

Classical algorithms based on heuristics, such as filtering, edge detection, derivatives, thresholding, time windows, and so forth, can be used to automatically detect features in ECGs [3]. Since the 1980s, algorithms have steadily increased in performance [4,5]. Machine learning, and particularly deep learning (DL), models have proven their superiority over classical algorithms in ECG analyses [6,7]. DL typically requires vast amounts of labeled training data. Publicly available annotated ECG datasets large enough to train DL models do not capture all possible ECG biases or distortions. The ECGs of patients located inside MRI scanners are distorted by a magnetohydrodynamic (MHD) effect, proportional to the intensity of the magnetic field [2,8]. In a strong magnetic field, MHD distortions, mostly caused by blood flowing through large vessels, induce elevated voltages in nearby conductors, which typically alter the shapes and amplitudes of T waves, as well as ECG baselines. An MHD-distorted ECG renders automated analyses unreliable, whether they are classical algorithms or DL architectures trained on non-distorted datasets [9]. Other distortions, caused by gradient switching and radiofrequency irradiation, are routinely removed by signal filtering and are out of the scope of the present work.

One publicly available ECG dataset acquired to study the impact of MHD distortions on the performance of a QRS detector was introduced by Krug et al. [8,10]. To address the scarcity of publicly available MHD-distorted ECG data, we propose a DL-based approach consisting of synthesizing realistic MHD distortion templates using a multivariate generative adversarial network (GAN) and adding them to a distortion-free ECG dataset. This results in datasets augmented with MHD-distorted ECGs, which can be used during the training phase of a DL-based R-peak detector for automatic ECG analyses.

## 2. Related Works

### 2.1. MHD Distortion Models

Current approaches for synthesizing MHD distortions are based on either empirical or mathematical/geometrical models [11]. Wavelet transformations and time and frequency domain analyses can distinguish between QRS complexes, magnetic field gradient distortions, and MHD effects [12]. Some approaches are limited to computing correlations between aortic blood flow and MHD distortions [13] or solving the Navier–Stokes equations [14]. These models poorly match the actual phenomenon. A more advanced model uses a finite-element representation of aortic blood flow, bi-domain equations in the heart, and electrical diffusion through the torso [15]. Using 4D MRI phase-contrast measurements of aortic blood flow [11], synthesized MHD distortions were compared to the measured ones, and showed different morphologies in the precordial leads.

### 2.2. Data Augmentation and Synthesis Techniques

DL architectures have been applied to a large variety of medical data analysis tasks [16,17]. Training datasets are often small and suffer from class imbalance issues [18], which can lead to overfitting and reduce the generalization capacity of the DL model, i.e., how well the model would perform on unseen data. Regularization strategies can be applied to address this issue, including reducing model complexity, introducing early stopping criteria or a penalty term to the loss function, using ensemble learning by combining several independent models, or using more data [19]. Augmenting the volume of training data is commonly carried out by applying mathematical, statistical, or random transformations. Time-series data synthesis techniques have been categorized as follows [20,21]:

#### 2.2.1. Transformation Methods

Random transformations can be applied to the amplitude (e.g., jittering, flipping, scaling, magnitude warping), time (e.g., slicing, permutation, time warping, time masking), or frequency (e.g., frequency warping, frequency masking, Fourier transform, spectrogram augmentation) [22].

#### 2.2.2. Pattern-Mixing Methods

Combining patterns to synthesize new data can be categorized as amplitude (e.g., averaging and interpolation, deviation from the mean), time (e.g., guided warping), frequency (e.g., equalized mixture data augmentation, stochastic feature mapping), or multiple domains (e.g., suboptimal element alignment averaging, barycentric averaging). For instance, Zahid et al. added a wandering baseline and motion artifacts to ECG segments containing one or more arrhythmic beats to improve the performance of an R-peak detector [23].

#### 2.2.3. Decomposition Methods

Decomposing time-series signals by extracting features or key patterns (e.g., independent component analysis, seasonal-trend decomposition using locally estimated scatterplot smoothing, empirical mode decomposition) can be used to train biometric systems more effectively. For instance, Soler et al. presented a Gaussian mixture model that synthesized cardiac cycles with ischemic alterations [24].

### 2.3. Generative Methods and Deep Generative Models

Generative methods based on sampling time series from feature distributions are classified as statistical models (e.g., Gaussian trees, posterior sampling, local and global trends, generating time series) and deep generative models (DGMs) [25].

Statistical models capture the dynamic distribution or probabilistic representation of a real-world dataset to synthesize new samples [26]. For instance, Que et al. synthesized the 12-lead ECG during myocardial infarction using a multi-scale ECG generative simulation model [27].

Based on the application of DL models trained to approximate real data distributions [28,29], DGMs can generate realistic multivariate time series that mimic the characteristics of real data [30]. Increasingly used to analyze ECG data [31,32,33,34,35,36], they comprise different DL architectures such as long short-term memory (LSTM), convolutional neural networks (CNNs), or recurrent neural networks (RNNs) [37].

**Figure 2:**
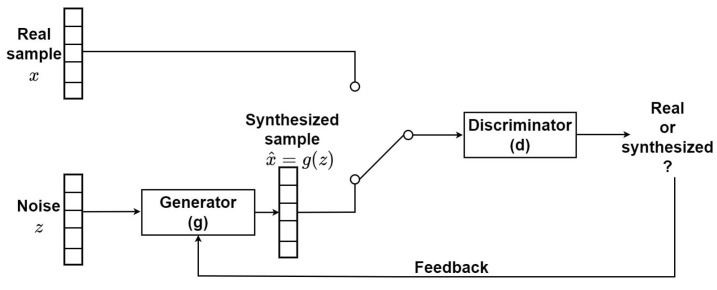
Block diagram of a GAN.

GANs are based on an adversarial training approach, i.e., to jointly optimize two neural networks (generator and discriminator) while they compete with each other [38]. The generator creates fake data samples by incorporating feedback from the discriminator, which classifies these samples as real or fake (see Figure 2).

In [39], numerous GANs were proposed to synthesize ECG data and improve the performance of ECG classifiers. Adib et al. compared the performances of five different GAN architectures, demonstrating the improved performance of ECG classifiers [40].

## 3. Materials and Methods

### 3.1. Datasets

#### 3.1.1. INCART

This is a high-quality dataset containing data on 75 patients with suspected coronary artery disease, recorded using regular Holters at a frequency of 257 Hz, which was released on the PhysioNet Website in 2008 [41]. The total number of annotated R-peaks is 175,907, for a total duration of 262,575 s [42,43].

#### 3.1.2. Getemed

This contains MHD-distorted ECGs from nine subjects placed inside a 3T MRI scanner (Magnetom Skyra, Siemens, Erlangen, Germany), without gradient switching or radiofrequency irradiation, in free breathing, recorded using a regular Holter (Getemed AG, Teltow, Germany) at 1024 Hz. The dataset was released on the PhysioNet Website in 2021 [41]. The recordings were available in head-first (HF) and feet-first (FF) positions for subject #1 and subjects #5 to #9 [8,10]. Ten recordings, from subject #1 and subjects #5 to #8, were used for data synthesis, with a total number of 3209 (resp. 2763) R-peaks in HF (resp. FF), for a total duration of 2967 s (resp. 2551 s). Recordings from subjects #2 to #4, only available in FF, were not used. The HF recording from subject #9 was used for testing. R-peaks were annotated by physicians or ECG experts.

#### 3.1.3. Schiller

This is a reconstructed dataset containing 12-lead MHD-distorted ECGs [44] from 3 subjects in HF acquired in an idle 3T MRI scanner (Magnetom Prisma, Siemens, Erlangen, Germany) using a prototype sensor (Schiller AG, Baar, Switzerland) at 1000 Hz. A total of 69 R-peaks were manually annotated by an ECG expert, for a total duration of 62 s.

#### 3.1.4. Siemens

This dataset contains 4-lead MHD-distorted ECGs from 3 patients in HF, acquired in a 3T MRI scanner (Magnetom Trio, Siemens, Erlangen, Germany) by the physiological unit of the equipment. A total of 51 R-peaks were manually annotated by an ECG expert, for a total duration of 52 s.

The INCART and Getemed 12-lead ECG datasets were used for data synthesis and R-peak detector training, considering R-peak annotations from lead II. Subject #9 from the Getemed dataset (a recording unseen during data synthesis and training) and the Schiller and Siemens datasets were used for testing.

### 3.2. Data Synthesis Pipeline

#### 3.2.1. MHD Distortion Input Database Preparation

**Figure 3:**
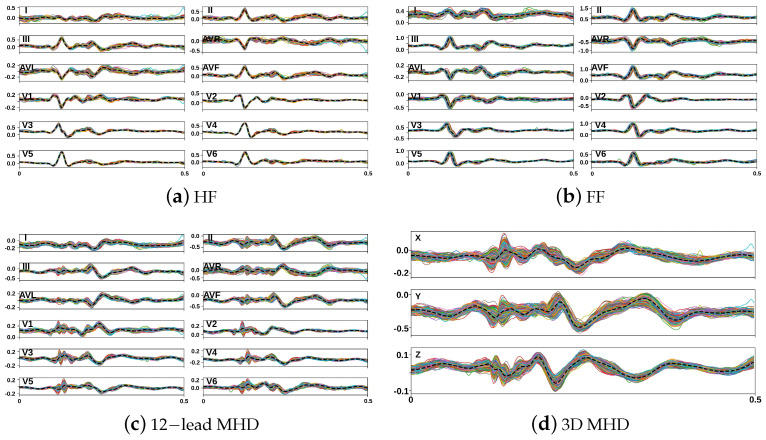
MHD distortion input database. (**a**,**b**) 12—lead ECG segments; (**c**) 12—lead MHD distortions; and (**d**) 3D (X, Y, and Z) MHD distortion segments extracted from subject #1 of the Getemed dataset. Dashed black line = median.

Raw 12-lead MHD-distorted ECG recordings (in HF and FF) from the Getemed dataset were resampled to 500 Hz. A bandpass filter with an order of 3 and a frequency range of [0.05, 150] and then a notch filter and a high-pass filter set to 0.05 Hz were applied to eliminate power-line interference and remove baseline wandering. Equal-sized segments of 0.5 s were extracted, placing labeled R-peaks at 25% of each 250-sample segment (see Figure 3a,b).

A total of 3204 (HF) and 2761 (FF) segments were obtained. A total of 2715 MHD distortion segments were obtained using the formula $(ECG_{HF}-ECG_{FF})/2$ [11]. Figure 3c shows the MHD distortion segments obtained from subject #1. Subsequently, the 12-lead MHD distortion segments were converted into a 3D vectorcardiogram (VCG) using the Kors regression transformation [45], which was then normalized (see Figure 3d). This constituted the MHD distortion input database.

#### 3.2.2. Synthesis of Realistic MHD Distortion Templates and ECG Dataset Augmentation

A GAN, characterized by an RNN-based generator and a CNN-based discriminator, was fed with the MHD distortion input database. Detailed parameters of the architecture of the proposed GAN are provided in Table 1.

The discriminator consisted of four convolutional layers (Conv1D) with the numbers of filters set to 32, 64, 128, and 256. All layers had a kernel size of 16, a stride of 1, the same padding, and a LeakyReLU activation function. A 1D MaxPooling layer was added after the LeakyReLU activation function in the 2nd and 4th convolutional layers. A flatten layer, followed by a Dense layer with a Sigmoid activation function, succeeded the convolutional layers, resulting in a single unit as output. The discriminator featured 706,017 trainable parameters and required 2.4 MB of GPU memory.

The generator started with a Dense layer, initialized with weights from a normal distribution with a standard deviation of 0.02. The Dense layer was followed by one BiLSTM layer with 24 hidden units, utilizing the sum as the merge mode, and employing a Tanh activation function. The BiLSTM layer was followed by a dropout layer with a drop rate of 50% and a Dense layer with a Tanh activation function. The generator featured a small number of trainable parameters (67,817) compared to other DL models and required a GPU memory of 1.7 MB.

**Figure 4:**
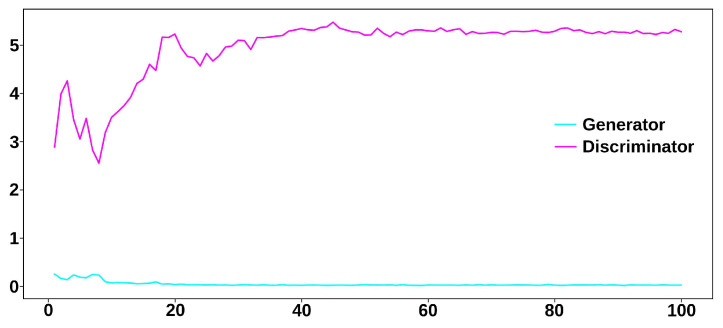
GAN loss curves.

The discriminator and generator used binary cross-entropy as the loss function and Adam as the optimizer, with exponential decay rates for the 1st and 2nd moment estimates set at 0.5 and 0.9, respectively. The learning rates were initially set at 0.0002 and 0.0004 for the discriminator and generator, respectively, and were reduced by a factor of 2 every 5 epochs. The proposed GAN was implemented using Python 3.10.12 and Keras 2.12 and was trained on a Tesla T4 GPU with 16 GB of RAM. The dataset consisted of 2715 segments, each comprising 250 samples from the 3D MHD distortion input database. The training spanned 100 epochs, with a batch size of 32, and a latent space dimension of 250. The total training time was 1156 s. The loss curves showed successful training (see Figure 4) since the losses of the discriminator and generator did not diverge as training progressed.

**Figure 5:**
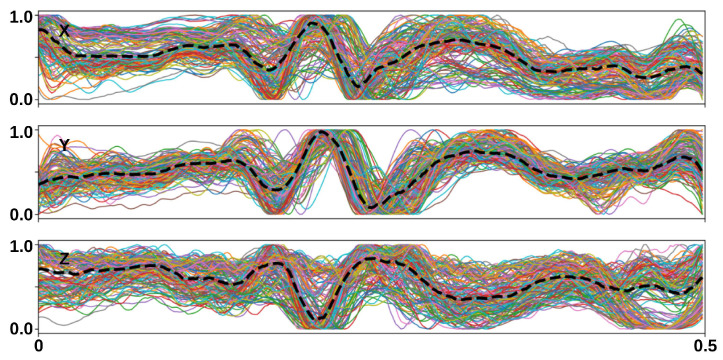
Synthesized MHD distortion templates using the GAN. The dashed black line = the median of the synthesized MHD distortion templates.

A database of 150 MHD distortion templates was generated using the proposed GAN. Similar morphologies were observed in the median curves between the measured (see Figure 3d) and synthesized MHD distortions (see Figure 5), with a closer match in the Y and Z components compared to the X component. The synthesized MHD distortion templates showed higher diversity.

**Figure 6:**
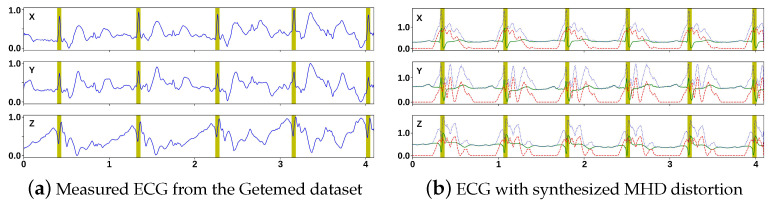
MHD-distorted ECG segments. (**a**) solid blue = measured ECG segment. (**b**) Solid green = INCART ECG segment. Dashed red line = synthesized MHD distortion template. Dotted blue line = synthesized MHD-distorted ECG segment. Vertical bars = annotated R-peak locations.

The MHD distortion templates were added to the normal and abnormal beats of the 75 INCART recordings after they were converted into 3D VCGs and then normalized to produce a larger dataset (see Figure 6b).

One MHD distortion template was repeated across a full recording at each heartbeat, respecting the R-peak locations. A total of 150 randomly selected MHD distortion templates were added to the 75 INCART ECG recordings to produce an augmented dataset of 225 ECG recordings containing MHD-distorted, as well as non-distorted, ECG recordings, called the GAN-augmented dataset.

The visual inspection demonstrated a good similarity between the measured and synthesized MHD-distorted ECG recordings (see Figure 6).

### 3.3. Evaluation Metrics

#### 3.3.1. Similarity Metrics

The maximum mean discrepancy (MMD) and dynamic time warping (DTW) were computed to assess the similarity between the synthesized MHD distortion templates and the input database used for training.

The MMD measures the dissimilarity between two probability distributions, *X* and $X^{\prime }$, by independently selecting samples from each distribution, according to Equation (1):

(1) $$\begin{array}{cc} MMD(X,X^{\prime }) & =\frac{1}{n(n-1)}\sum_{i=1}^{n}\sum_{j\neq 1}^{n}K\left(x_{i},x_{j}\right)-\frac{2}{nm}\sum_{i=1}^{n}\sum_{j=1}^{m}K\left(x_{i},x_{j}^{\prime }\right)\\ \; & +\frac{1}{m(m-1)}\sum_{i=1}^{m}\sum_{j\neq 1}^{m}e^{-{∥x_{i}-x_{j}^{\prime }∥}^{2}} \end{array}$$

where *K* denotes the Gaussian radial basis function kernel used to compute the pairwise distance between each pair of rows $x_{i}$ in *X* and $x_{i}^{\prime }$ in $X^{\prime }$. *n* and *m* correspond to the dimensions of *X* and $X^{\prime }$, respectively.

DTW measures the dissimilarity between two time series by computing the Euclidean distance between them after warping data along the temporal axis to align the series. DTW is defined according to Equation (2):

(2) $$DTW\left(X,X^{\prime }\right)=\sqrt{\sum_{(i,j)\in l}{∥x_{i}-x_{j}^{\prime }∥}^{2}}$$

where $l=[l_{0},\ldots ,l_{K}]$ denotes the optimal alignment path between *X* and $X^{\prime }$ that satisfies the following properties:

- *l* is a list of index pairs $l_{k}=\left(i_{k},j_{k}\right)$, with $0\leq i_{k}<n$ and $0\leq j_{k}<m$.
- $l_{0}=\left(0,0\right)$ and $l_{K}=\left(n-1,m-1\right)$.
- For all k>0, $l_{k}=\left(i_{k},j_{k}\right)$ is related to $l_{k-1}=\left(i_{k-1},j_{k-1}\right)$ as follows:
  ∘ $i_{k-1}\leq i_{k}\leq i_{k-1}+1$.
  ∘ $j_{k-1}\leq j_{k}\leq j_{k-1}+1$.

#### 3.3.2. Accuracy of a DL-Based R-Peak Detector Trained Using Augmented Data

Evaluating the performance of augmented data when training a DL-based R-peak detector is another way to measure the adequacy of synthesized MHD-distorted ECG databases in real-world applications. The DL-based model used for this evaluation was described by Mehri et al. [7], along with the metrics used to measure its accuracy: precision P (or positive predictive value), recall R (or sensitivity), and F1-score [5]. A tolerance of ±75 ms was set between the annotated R-peak locations and the detected locations when counting true positives (TPs), false positives (FPs), or false negatives (FNs). The DL-based R-peak detector was first trained using the 75 INCART ECG recordings without data augmentation, and then using the GAN-augmented dataset. It was applied to detect R-peaks in subject #9 from the Getemed dataset, as well as in the Schiller and Siemens datasets.

## 4. Results

The MMD and DTW values for the proposed GAN are summarized in Table 2. These metrics show that the GAN can generate 3D MHD distortion templates of good quality. The MHD distortion templates synthesized using the GAN achieved low MMD and DTW values, meaning a higher similarity with those of the input database. The lowest MMD and DTW values were observed in the Y and Z components, respectively.

**Figure 7:**
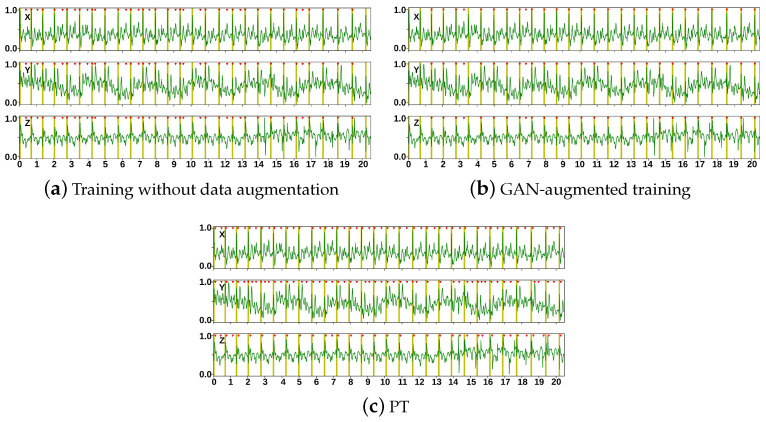
Qualitative results of a DL-based R-peak detector trained without data augmentation and with the GAN-augmented dataset, applied to subject #9 from the Getemed dataset. Results of the Pan–Tompkins (PT) algorithm are added as a reference. Detected R-peaks are marked with red triangles, whereas vertical bars mark the annotated R-peak locations.

The results from the R-peak detector trained with and without data augmentation are presented in Figure 7 and Table 3.

GAN-augmented training significantly reduced the number of FPs, leading to improved precision. The number of FNs was not increased after GAN-augmented training. Accordingly, GAN-augmented training performed significantly better than training without data augmentation. The overall gains in terms of the F1-score with GAN-augmented training were 15.3%, 0.66%, and 10.12% on the Getemed, Schiller, and Siemens datasets, respectively. Compared to the reference PT algorithm [46], the GAN performed better, except in the Schiller dataset, where the PT algorithm demonstrated better recall.

## 5. Discussion

In cardiac MRI, and particularly Cine-MRI, image quality is directly impacted by the accurate and timely detections of R-peaks. MRI pulse sequences employed to acquire k-space data are synchronized with R-peaks in a process known as triggering or gating [2]. ECG distortions, which occur when a patient lies inside an MRI scanner, make automated ECG analyses unreliable. Gradients or radiofrequency distortions are usually addressed by filtering, whereas the MHD effect is more challenging. ECG datasets used to train DL models do not typically include MHD distortions.

With the proposed approach, synthesized MHD-distorted ECG datasets can be used to efficiently train DL models. We demonstrated enhanced precision (i.e., a low number of false R-peak detections) in a state-of-the-art R-peak detector, which guarantees a more reliable ECG-triggered cardiac MRI. This approach could be used to build distorted ECG datasets from larger patient populations, featuring arrhythmia and other pathologies, thus overcoming the limitations of building ECG datasets from healthy volunteers. In this work, we used the INCART dataset, which is a large publicly available 12-lead ECG dataset featuring arrhythmia and other abnormalities.

GAN-augmented training significantly outperformed training based on a dataset without MHD distortions. The proposed approach has a high generalization capacity, with the results obtained on MHD-distorted ECG datasets differing from those used in the training phase. Through GAN-augmented training, the overall error was reduced by almost 4.48, 1.31, and completely eliminated, respectively, on three different datasets. The performance gains of the R-peak detections with GAN-augmented training were lower on the Schiller dataset compared to the Getemed and Siemens datasets. This can be explained by the specificities of the Schiller dataset. The Schiller dataset is composed of reconstructed 12-lead ECGs, which reduced MHD amplitudes by design [44]. As a result, training without GAN data augmentation, as well as the PT algorithm, performed well.

The GAN used in our approach is reputed for its ability to effectively synthesize high-quality time-series data [47,48]. We compared its performance to that of a variational autoencoder (VAE) model with a similar architecture and hyperparameters on the Getemed dataset. The results from the DL-based R-peak detector trained without data augmentation (w/o), with a GAN-augmented dataset, and with a VAE-augmented dataset are presented in Table 4.

GAN-augmented training outperformed VAE-augmented training. VAE-augmented training achieved a lower recall than training without data augmentation. VAE models suffer from mode collapse since they work by constraining the latent space distribution on an individual training example basis. Increasing the number of hidden units or layers, adding regularization terms such as weight decay, or using a stronger prior such as a dropout in the network architecture could resolve the mode collapse issue and improve the performance of the VAE model (i.e., allowing it to learn more complex distributions). GAN models can identify deeper insights from input data and generate higher and more realistic data compared to VAE models. They have a more complex supervised training process, requiring synchronization between their two components, the generator and discriminator, than VAE. This could explain why GAN training outperformed VAE training.

The stability of the training process of DGMs can be improved. For instance, parameters can be optimized by a loss function based on data quality metrics. Furthermore, the VAE and GAN-based approaches have the drawback of not reconciling the two conflicting objectives in generative modeling: traceability, and flexibility. Tractable models are able to easily fit data and evaluate but are unable to describe the structure of rich datasets. Flexible models are able to fit arbitrary structures in data but are computationally expensive during training and evaluation. Score-based diffusion models could potentially overcome these limitations. Our experiments were limited to a magnetic field strength of 3T and could be extended to other field strengths using an appropriate MHD distortion input database.

The primary area of application of this work could be to make R-peak detection more reliable in MHD-distorted ECG. It could also be generalized to other ECG features such as P or T waves. R-peak detection remains a preliminary step and conditions the rest of automated ECG analyses. State-of-the-art VCG-based approaches [49] are not fully reliable in magnetic fields of 3T or higher, forcing clinicians to move electrodes or use other techniques [50], potentially reducing MRI quality. In the worst case, the exam is not possible or interpretable by the physician. Reliable ECG-triggered cardiac MRI requires good precision, i.e., a low number of false R-peak detections, which was demonstrated by our approach. With the recent developments of edge devices, the inference time of a DL-based R-peak detector is on the order of a few ms; hence, it is compatible with the real-time requirements of triggered cardiac imaging.

## 6. Conclusions

In this article, we introduced an effective approach for synthesizing 3D MHD distortions using a multivariate GAN. The proposed approach consists of identifying, modeling, and reproducing the MHD distortions using the GAN model and adding them to available, distortion-free datasets to create additional training data, exposing DL models to a large variety of data, and hence building more reliable DL models. We conducted thorough experiments to show that high-quality 3D MHD-distorted ECG datasets can be generated based on publicly available ECG datasets and a limited number of MHD distortions as input. We also provided a comprehensive and in-depth case study to demonstrate that the synthesized dataset can be used to effectively train a DL-based R-peak detector and increase its precision in MHD-distorted ECG analysis. The proposed approach showed high performances in terms of both precision and recall on different MHD-distorted ECG datasets that were unseen during the training phase.

## Figures and Tables

**Table 1:** GAN parameters.

	Layer	Output Shape	# of Parameters
Discriminator	Conv1D	(32, 250, 32)	1568
	LeakyReLU	(32, 250, 32)	0
	Conv1D	(32, 250, 64)	32,832
	LeakyReLU	(32, 250, 64)	0
	MaxPooling1D	(32, 125, 64)	0
	Conv1D	(32, 125, 128)	131,200
	LeakyReLU	(32, 125, 128)	0
	Conv1D	(32, 125, 256)	524,544
	LeakyReLU	(32, 125, 256)	0
	MaxPooling1D	(32, 62, 256)	0
	Flatten	(32, 15872)	0
	Dense	(32, 1)	15,873
Generator	Dense	(32, 1, 250)	62,750
	Reshape	(32, 250, 1)	0
	Bidirectional	(32, 250, 24)	4992
	Dropout	(32, 250, 24)	0
	Dense	(32, 250, 3)	75

**Table 2:** Evaluation of the quality of the MHD distortion templates synthesized by the GAN. μ = average; σ = standard deviation.

	MMD	DTW
X	0.85	121.27
Y	0.56	155.66
Z	0.97	104.78
μ±σ	0.79 ± 0.28	127.24 ± 35.97

**Table 3:** Quantitative results of a DL-based R-peak detector trained without data augmentation (w/o) and with the GAN-augmented dataset. Results of the Pan–Tompkins (PT) algorithm are added as a reference.

Dataset	Method	# of R-Peaks	TPs	FPs	FNs	P (%)	R (%)	F1 (%)
Getemed	w/o	887	865	402	22	68.27	97.52	80.31
	GAN		872	65	15	93.06	98.31	95.61
	PT		817	560	70	64.37	92.11	74.41
Schiller	w/o	69	67	1	2	98.72	96.08	97.26
	GAN		67	0	2	100.00	96.08	97.92
	PT		68	1	1	98.49	98.69	98.54
Siemens	w/o	51	46	2	5	91.67	88.68	89.88
	GAN		51	0	0	100.00	100.00	100.00
	PT		49	16	2	74.91	92.59	81.72

**Table 4:** Quantitative results on the Getemed dataset of a DL-based R-peak detector trained without data augmentation (w/o) and with GAN-augmented and VAE-augmented datasets.

Method	P (%)	R (%)	F1 (%)
w/o	68.27	97.52	80.31
GAN	93.06	98.31	95.61
VAE	81.78	87.03	84.32

## Data Availability

The INCART and Getemed datasets used in this research are publicly available on PhysioNet at https://physionet.org/about/database accessed on 12 September 2023. The Schiller dataset is potentially available upon request, pending an internal review of such a request. Belonging to the University Hospital of Dijon, the Siemens dataset cannot be shared publicly without the permission of this center. However, it is available upon request.

## Abbreviations

The following abbreviations are used in this manuscript:

BLSTM	Bidirectional long short-term memory
CNN	Convolutional neural network
DGM	Deep generative models
DL	Deep learning
DTW	Dynamic time warping
ECG	Electrocardiogram
GAN	Generative adversarial network
HF	Head first
FF	Feet first
FN	False negative
FP	False positive
LSTM	Long short-term memory
MHD	Magnetohydrodynamic
MMD	Maximum mean discrepancy
MRI	Magnetic resonance imaging
RNN	Recurrent neural network
TP	True positive
VAE	Variational autoencoder
VCG	Vectorcardiogram
